# Noncoding RNAs in pediatric brain tumors: Molecular functions and pathological implications

**DOI:** 10.1016/j.omtn.2021.07.024

**Published:** 2021-08-08

**Authors:** Shaohuai Chen, Xiangyang Deng, Hansong Sheng, Yuxi Rong, Yanhao Zheng, Yusong Zhang, Jian Lin

**Affiliations:** 1 Department of Neurosurgery, The Second Affiliated Hospital and Yuying Children’s Hospital of Wenzhou Medical University, Wenzhou, China

**Keywords:** lncRNAs, miRNAs, circRNAs, brain tumor, therapy

## Abstract

Brain tumors are common solid pediatric malignancies and the main reason for cancer-related death in the pediatric setting. Recently, evidence has revealed that noncoding RNAs (ncRNAs), including microRNAs (miRNAs), long ncRNAs (lncRNAs), and circular RNAs (circRNAs), play a critical role in brain tumor development and progression. Therefore, in this review article, we describe the functions and molecular mechanisms of ncRNAs in multiple types of cancer, including medulloblastoma, pilocytic astrocytoma, ependymoma, atypical teratoid/rhabdoid tumor, glioblastoma, diffuse intrinsic pontine glioma, and craniopharyngioma. We also mention the limitations of using ncRNAs as therapeutic targets because of the nonspecificity of ncRNA targets and the delivery methods of ncRNAs. Due to the critical role of ncRNAs in brain oncogenesis, targeting aberrantly expressed ncRNAs might be an effective strategy to improve the outcomes of pediatric patients with brain tumors.

## Graphical abstract

**Figure:**
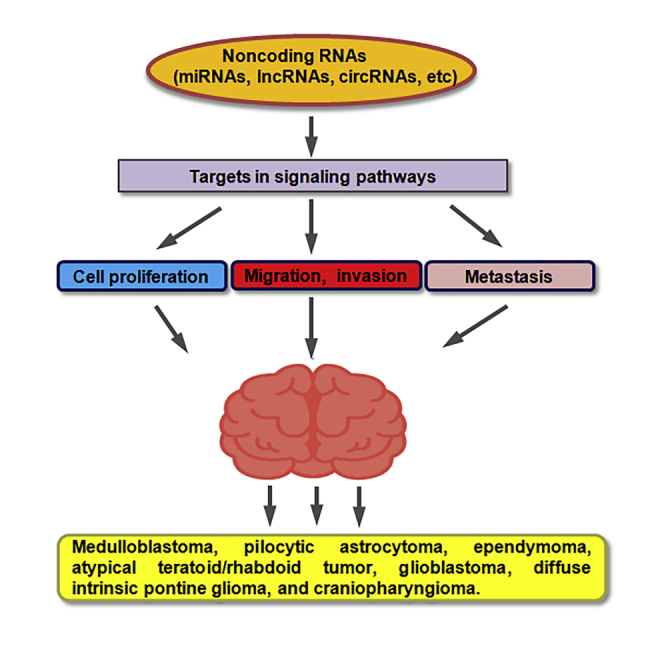

## Teaser

In this review article, the authors describe the functions and molecular mechanisms of ncRNAs, such as miRNAs, lncRNAs, and circRNAs, in pediatric brain malignancies, including medulloblastoma, pilocytic astrocytoma, ependymoma, atypical teratoid/rhabdoid tumor, glioblastoma, diffuse intrinsic pontine glioma, and craniopharyngioma.

## Introduction

Brain tumors are the most frequent solid pediatric malignancies and the main cause of cancer-related death in the pediatric setting.^1^, ^2^, ^3^ The World Health Organization (WHO) classification of tumors of the central nervous system (CNS) includes diffuse astrocytic and oligodendroglial tumors, other astrocytic tumors, ependymal tumors, other gliomas, choroid plexus tumors, neuronal and mixed neuronal-glial tumors, tumors of the pineal region, embryonal tumors, and tumors of the cranial and paraspinal nerves.^4^ Medulloblastoma (MB), pilocytic astrocytoma (PA), ependymoma (EPN), and atypical teratoid/rhabdoid tumor (ATRT) have become important health problems with adverse medical consequences in children. Treatment of these tumors usually requires a multimodality approach that includes surgical intervention, radiotherapy, and chemotherapy. Nonetheless, since the developing nervous system is highly susceptible to damage from these conventional therapeutic strategies and resistance to therapeutic drugs can occur, the treatment of malignant pediatric brain tumors still faces difficult challenges.^2^^,^^5^

Great advances in molecular genetics, epigenetics, and cellular biology have provided a wealth of clinically and biologically significant insights into these deadly childhood diseases, potentially enabling the development of more effective and less toxic treatment strategies. Noncoding RNAs (ncRNAs) are emerging as essential regulators of diverse biological processes, including human oncogenesis and tumor progression. Recently, ncRNAs have attracted increasing attention because they lack the capacity to encode proteins.^6^, ^7^, ^8^ Based on a 200-nt cutoff in mature transcript length, ncRNAs are commonly divided into small ncRNAs (sncRNAs, 18–200 nt) and long ncRNAs (lncRNAs, >200 nt).^9^ To date, several kinds of sncRNAs have been well defined, including microRNAs (miRNAs), small nuclear RNAs (snRNAs), piwi-interacting RNAs (piRNAs), and small nucleolar RNAs (snoRNAs).^10^, ^11^, ^12^ According to their genomic localization and evolutionary lineage, lncRNAs are classified as long intergenic RNAs (lincRNAs), antisense RNAs, sense intronic RNAs, enhancer RNAs (eRNAs), and pseudogenes.^13^ As a new research hotspot in the field of miRNAs and lncRNAs, many circular RNAs (circRNAs) have also been observed in and associated with distinct cancers.^14^^,^^15^ These molecules play a pivotal role in all critical biological processes controlling various levels of gene expression via epigenetic modification, transcription, RNA splicing, and scaffold assembly.^7^^,^^16^^,^^17^ Moreover, their aberrant expression is confirmed to be involved in oncogenesis and disease progression, making them a new class of potential treatment targets with broad applicability.

With the number of ncRNAs steadily increasing due to the rapid development of high-throughput sequencing and bioinformatics technologies, it is necessary to summarize the current research progress on ncRNAs in pediatric brain tumors. A deeper understanding of the function and mechanism of action of ncRNAs will drive the development of new treatment strategies for pediatric neuro-oncology. Herein, we conducted a systematic literature review regarding the deregulation of ncRNAs with a focus on miRNAs, lncRNAs, and circRNAs, as well as the pathological implications for the biology of pediatric CNS tumors to provide insights into the diagnostic, prognostic, and therapeutic potential of ncRNAs (Tables 1 and 2).

**Table 1:** lncRNAs in brain tumors

LncRNAs	Expression	Phenotype	Downstream targets	Refs.
Gm15577	↓	tumorigenesis	Negr1	^18^
CRNDE	↓	proliferation, apoptosis, migration, invasion	miR-29c-3p	^19^^,^^20^
linc-NeD125	↑	proliferation, migration, invasion	miR-19a-3p, miR-19b-3p, miR-106a-5p, CDK6, MYCN, SNCAIP	^21^
Nkx2-2as	↓	tumorigenesis	miR-103/107/BTG2/Tis21/PC3, miR-548 m/LATS1/2	^22^
UCA1	↑	proliferation, migration	PI3K/AKT pathways	^23^
CCAT1	↑	proliferation, migration	MAPK pathway	^24^
LOXL1-AS1	↓	growth, migration	PI3K/AKT	^25^
TP73-AS1	↓	survival, migration, proliferation	miR-494-3p, EIF5A2	^26^^,^^27^
HOTAIR	↑	proliferation, migration, invasion, apoptosis, EMT	miR-1/miR-206-YY1, HOXA9, miR-125, miR-219	28, 29, 30, 31, 32, 33, 34, 35, 36, 37
LINC00899	↑	invasion, migration	RBL2	^38^
TRERNA1	↑	EMT	Snail	^39^
MALAT	↑	proliferation, progression	miR-155, miR-199a, FBXW7, ZHX1	40, 41, 42, 43, 44
DGCR5	↓	EMT	epithelial markers	^45^

**Table 2:** circRNAs in glioblastoma

CircRNAs	Expression	Phenotype	Downstream targets	Refs.
circ-FBXW7	↓	proliferation, cell cycle	FBXW7-185aa	^46^
circ-SHPRH	↓	tumorigenicity	SHPRH-146aa	^47^
circSMARCA5	↓	migration	SRSF1/SRSF3/PTB	^48^^,^^49^
hsa_circ_0008344	↑	proliferation, migration, invasion, colony formation, apoptosis	miR-433-3p/miR-450b-3p	^50^
circNT5E	↑	proliferation, migration, invasion	miR-422a	^51^
circPINTexon2	↓	proliferation	PINT87aa	^52^
circMMP9	↑	proliferation, invasion, metastasis	miR-124	^53^
circ-0029426	↑	proliferation, migration, invasion, apoptosis	miR-197	^54^
circ-0074027	↓	growth, invasion	miR-518a-5p/IL17RD	^55^
circ-0067934	↑	proliferation, metastasis	PI3K-AKT pathway	^56^
circ-0001730	↑	proliferation, invasion	miR-326/Wnt7B axis	^57^
circMTO1	↓	proliferation	miR-92/WWOX	^58^
circ-AKT3	↓	proliferation, radioresistance	AKT-Thr308	^59^
circ-PITX1	↑	progression	miR-379-5p/MAP3K2	^60^
circFOXO3	↑	progression	miR-138-5p, miR-432-5p	^61^
circ-0001801	↑	proliferation, migration, invasion, EMT	miR-628-5p/HMGB3 axis	^62^
circ-EPB41L5	↑	progression	EPB41L5/p-AKT	^63^
circENTPD7	↑	proliferation, motility	miR-101-3p/ROS1	^64^

## PAs

PAs are the most frequent CNS neoplasms in childhood, accounting for approximately 20% of all pediatric brain tumors with an average annual age-adjusted incidence rate of 0.8.^65^, ^66^, ^67^ PAs are usually considered relatively benign (WHO grade I) tumors with a 10-year survival rate >90%.^65^ These tumors occur throughout the CNS, with the cerebellum being the most frequent location in the pediatric setting. Biologically, alterations in the mitogen-activated protein kinase (MAPK) signaling pathway, KIAA1549-BRAF fusions, and neurofibromatosis type 1 (NF1) syndrome have been shown to impact PA development.^68^, ^69^, ^70^

### miRNAs and lncRNAs in PAs

Compelling evidence has identified a number of aberrantly expressed miRNAs in PAs, which has aided the discovery of novel diagnostic methods and effective treatments for this type of tumor. A survey of miRNA expression demonstrated that miR-142-5p and miR-25 were significantly upregulated in PAs compared to normal tissue, while miR-129 was strongly downregulated. Compared to those in other CNS pediatric tumors (ATRT, EPN, MB, and glioblastoma), multiple miRNAs, including miR-93 and miR-106b, were observed to be downregulated, whereas several miRNAs, such as miR-432 and miR-34a, were found to be upregulated in PSs.^71^ Dysregulated expression levels of a subset of miRNAs, including decreased expression of miR-129 and miR-124 and overexpression of miR-21, were also observed in PAs. In addition, miR-650 and miR-1276 levels were increased, while miR-744∗ and miR-187∗ levels were decreased, in NF1-associated tumors among the PA subgroups.^72^ Similarly, miR-15 and miR-24-1 levels were reported to be decreased in PAs.^73^

Jones et al.^74^ also found that the Xq26.3 cluster, miR-224, miR-146a, miR-34a, and the miR-106a∼miR-363 cluster were upregulated, while miR-124, miR-129, and miR-218 were downregulated. Predicted targets of differentially regulated miRNAs frequently include components of the extracellular signal-regulated kinase (ERK)/MAPK and nuclear factor κB (NF-κB) signaling pathways. Another study identified 88 miRNAs that were expressed to different degrees between PA and cerebral white matter samples.^75^ PA samples had the most downregulated miRNAs regulating classical pathways of tumorigenesis, while the most overexpressed miRNAs were associated with pathways such as focal adhesion, the p53 signaling pathway, and gliomagenesis. High expression of miR-34a-5p and miR-144-3p and low expression of miR-630 and miR-139-3p were further confirmed by qRT-PCR.^75^ Yuan et al.^76^ also demonstrated that miR-125 family members were downregulated in PAs compared to nonneoplastic brain, and overexpression of miR-125b in pediatric low-grade glioma decreased cell growth and invasion and induced apoptosis. Furthermore, one study analyzed the expression of lncRNA HOTAIR in five pediatric tumor types and found higher expression of this gene in juvenile PAs.^28^

## MBs

MBs, as embryonal tumors of the cerebellum, account for approximately 20% of the total brain tumors in this patient population.^67^^,^^77^^,^^78^ Advances in treatment with neurosurgery, radiation therapy, and high-dose chemotherapy have significantly improved the survival rate of these patients. However, long-term sequelae, including neurocognitive, neuroendocrine, and psychosocial deficits caused by intensive therapies administered to the developing brain, remain challenging. Therefore, more effective molecular-targeted strategies with less toxicity are urgently needed to be developed for this disease.^67^^,^^77^

Considering the nature of its molecular heterogeneity, a great deal of genomic research has helped classify MB into four subgroups: Wingless (WNT), Sonic hedgehog (SHH), group 3, and group 4.^79^ These subgroups have different genetic alterations, clinical features, and results. WNT tumors, characterized by activated WNT signaling, occur primarily in children older than 3 years of age and exhibit a balanced sex ratio. These tumors have few metastases and have a favorable prognosis, with a 5-year survival rate of more than 95%.^77^^,^^80^, ^81^, ^82^ Although WNT MB has been considered a largely homogeneous cluster, the extent of heterogeneity within the subgroups was further analyzed. WNT-α and WNT-β are two molecular subtypes that have been identified, and their differences are age at diagnosis and frequency of monosomy 6.^83^ SHH tumors are characterized by activation of the SHH pathway and have a 5-year survival rate of 75%, which is worse than that of WNT patients. This MB subtype presents a bimodal age distribution, with most cases being diagnosed in both infants and adults. Recently, four molecular subtypes of SHH MB, that is, SHH-α, SHH-β, SHH-γ and SHH-δ, have been described based on gene expression data and DNA methylation.^78^^,^^83^ A defining feature of group 3 MB is the high level of MYC amplification, which accounts for approximately 25% of MBs with the worst prognosis. Group 4 MB is the least known of the MB subgroups, and its molecular profiles are not as well characterized. Many different subtypes of groups 3 and 4 MBs have also been proposed.^78^^,^^84^, ^85^, ^86^

### Role of miRNAs in MBs

Along with increasing research on the ncRNA domain, increasing evidence supports the important roles of various miRNAs in MB. In this respect, the expression of miR-124 was reported to be significantly decreased in MB cells and tumor tissues.^87^ Further *in vivo* and *in vitro* experiments demonstrated that miR-124, acting as a tumor suppressor, inhibited tumor cell growth by targeting cyclin-dependent kinase 6 (CDK6), which is a member of the family of serine-threonine kinases that promotes cell cycle progression.^87^^,^^88^ SLC16A1 overexpression could promote cell proliferation and was found to be regulated by miR-124 in MB.^89^ In addition, the nuclear receptor Nur77, encoded by the *NR4A1* gene, is commonly upregulated in MB and leads to a proliferative state that promotes cancer progression, and it was also reported to be another target of miR-124.^90^ In addition, miR-199b-5p expression was found to be downregulated in MB via epigenetic methylation.^91^^,^^92^ miR-199b-5p expression can cause specific damage to the cancer stem cell (CD133^+^) population through negative regulation of the transcription factor HES1, which is a principal Notch-responsive factor.^92^ Its obvious downregulation in metastatic MBs also suggests a potential silencing mechanism that acts through epigenetic or genetic alterations.^92^ Moreover, as a marker of MB tumor-propagating cells, CD15 is an additional direct target of miR-199b-5p.^91^

As a Notch signaling pathway regulator, miR-34a can regulate DLL1, Jagged1, Notch1, and Notch2. Re-expression of miR-34a in MB cell lines strongly inhibited cell cycle progression, proliferation, survival, migration, and invasion, and it caused apoptosis and downregulated the expression of miR-34a targets, including c-Met, SIRT1, and MYCN proteins.^93^, ^94^, ^95^ miR-34a deficiency also accelerated MB genesis *in vivo*.^95^ In addition, it has been reported that this miRNA could render MB cells more sensitive to chemotherapeutic agents through the adjustment of MAGE-A and p53.^96^^,^^97^ Comparatively, with regard to chemotherapeutic resistance, SPARC-mediated cisplatin resistance could be regulated by miR-let-7f-1 through the let-7f-1 miRNA/HMGB1 axis in MB cells.^98^ Furthermore, one study revealed the roles of miR-584-5p in the regulation of DNA repair, microtubule dynamics, and stemness in MB, the potentiation of vincristine, and the radiation response via the miR-584-5p/HDAC1/eIF4E3 axis.^99^ In addition, miR-31 activated the phosphatidylinositol 3-kinase (PI3K)/AKT and NF-κB pathways, contributing to cisplatin resistance, and inducing cell growth, invasion, and migration in MB cells.^100^

miR-9 and miR-125a promote cell growth arrest and apoptosis in MB cells through modulation of the pro-proliferative truncated TrkC (t-TrkC) isoform.^101^ It has been verified that miR-9 is a methylation-silenced tumor suppressor contributing to disease pathogenesis through regulation of HES1 oncogenic activity.^102^ A survey of miRNA expression indicated that, relative to their expression in normal brain tissue, multiple miRNAs, including miR-216 and miR-340, were upregulated, whereas several miRNAs, such as miR-146b and miR-23a, were downregulated in MB.^71^ A high-throughput miRNA microarray was performed, and some miRNAs were confirmed to be downregulated in MB, including miR-17, miR-100, miR-106b, and miR-218. The predicted target genes are involved in MB development.^103^ miR-217, miR-216, miR-183, miR-182, and miR-96 were found to be upregulated in tumor tissue through analysis of the GEO miRNA expression database, whereas miR-383, miR-206, miR-138, miR-128a/b, and miR-133b were identified to be downregulated.^104^ Inhibition of miR-217 was further confirmed to induce apoptosis and reduce migration and invasion in MBs.^105^ miR-128a was found to inhibit cell growth by targeting Bmi-1, resulting in increased steady-state levels of superoxide and cellular senescence in MB.^106^

Upregulation of miR-383 expression decreased PRDX3 expression, leading to cell apoptosis and inhibition of proliferation in MBs.^107^ miR-31 was reported to suppress MB tumorigenesis by negatively regulating DNA replication via MCM2.^108^ Arhgef6, which is upregulated in human MBs and involved in mediating experimental medulloblastomagenesis, was repressed by miR-135a.^109^ Transient overexpression of miR-367, which is upregulated by OCT4 in MB cells, conspicuously enhanced proliferation, invasion, and generation of neurosphere-like structures, which are enriched in CD133-expressing cells.^110^ Additionally, miRNA-10b contributed to MB tumorigenesis with Bcl-2 as a mediator of the effects on MB cell survival.^111^ Restoration of miR-30a, miR-221-3p, and miRNA-4521 expression inhibited the proliferation, clonogenic potential, and tumorigenicity of MB cells.^112^, ^113^, ^114^ Moreover, miR-378 downregulated the activity of UHRF1, leading to modulating MB cell proliferation and apoptosis.^115^ The miR-512-2 gene was deleted in one-third of MBs associated with overexpression of MYCC, which was significantly correlated with tumor anaplasia and poor prognosis.^116^

Several studies reported that increased expression of miR-21 could promote cancer cell migration in MB.^117^^,^^118^ Moreover, miR-21 was proven to act on PDCD4, regulating the expression of multiple invasion- and metastasis-related proteins, including MAP4K1, JNK, E-cadherin, and TIMP2.^117^ A link with the STAT3/miR-21/PIAS3 circuitry that could mediate MB progression and metastasis was also established.^118^ Similarly, miR-182 was found to help accelerate leptomeningeal metastatic dissemination in non-SHH MBs.^119^ One group reported that miR-192 modulated the expression of DHFR, integrins, and CD47 to modulate cell proliferation and anchoring, resulting in the suppression of leptomeningeal dissemination in MBs.^120^ A miRNA-1280/JAG2 network was found to be associated with MB metastatic dissemination and patient outcomes.^121^ Expression of miR-210 might promote metastasis, and miR-206 had a suppressive role in MB viability and migration by targeting LASP1 and OTX2.^122^, ^123^, ^124^ Moreover, re-expression of miR-218 decreased cell growth, cell colony formation, cell migration, invasion, and tumor sphere size in MBs by directly regulating SH3GL1, and CDK6, RICTOR, and cathepsin B (CTSB) might be additional targets.^125^^,^^126^ The absence of miR-219 could enhance cell proliferation, invasion, and migration through regulation of CD164.^127^^,^^128^ PTEN is a direct target of miR-106, and deletion of miR-106b inhibits cell proliferation, migration, invasion, and tumor sphere formation through suppression of PTEN.^129^

Notably, efforts were made to characterize four consensus molecular subgroups using miRNA profiles. Many miRNAs, such as miR-193a, the miR-224/miR-452 cluster, the miR-182/miR-183/miR-96 cluster, and miR-148a, were found to be overexpressed in WNT MB. Overexpression of miR-193a and miR-224, which are upregulated WNT pathway-specific miRNAs, was verified to inhibit proliferation, increase radiation sensitivity, and inhibit anchorage-independent growth of MB cells.^130^ Upregulation of miR-193a-3p, miR-224, miR-148a, miR-23b, and miR-365 in WNT subgroup tumors was further validated in a study using 103 MB patients. This study indicated that miR-10b was increased in WNT MBs, followed by group 3 subtypes, while miR-182, miR-135b, and miR-204 were downregulated in SHH variants. miR-376a had higher expression in group 4 MB than in group 3 subtypes, and miR-592 was upregulated in group 4 MB. In addition, miR-135b was detected at low expression levels in groups 3 and 4 MBs.^131^ Accordingly, miR-148a might contribute to downregulating metastatic incidence and upregulating survival of WNT MB. High expression of miR-148a was confirmed to inhibit proliferation, clonogenic potential, invasion potential, and tumorigenicity of MB cells by targeting neuropilin 1.^132^ miR-499a, a candidate tumor suppressor gene, was found to be a potential marker for WNT MB.^133^^,^^134^

A study confirmed that downregulation of miR-125b, miR-324-5p, and miR-326 could be associated with the modulation of endogenous SHH target genes, including *Smo*, *Gli1*, and *Pitch*, and re-expression of these miRNAs in MB cells suppressed progenitor and tumor cell growth.^135^^,^^136^ Among them was miR-326, which is associated with the development of tumor stem cells derived from SHH MB. Expression of miR-326 was further confirmed to inhibit the Hh/Gli signaling pathway, impairing MB cell proliferation and self-renewal by negatively regulating Smo and Gli2.^137^ Three miRNA clusters, i.e., miR-183∼96∼182, miR-17-92, and miR-106b∼25, functionally collaborated with the SHH signaling pathway in MB development in mice.^137^, ^138^, ^139^, ^140^, ^141^, ^142^ miR-183∼96∼182 was further identified as protumorigenic in MYC-driven MB through suppression of apoptosis, modulation of the mTOR pathway, and control of motility.^143^ Analysis of human MBs also showed three miR-17∼92 cluster miRNAs, and the SHH signaling pathway could be constitutively activated by miR-92, miR-19a, and miR-20 overexpression.^140^ Inhibition of MB cell proliferation and tumor growth *in vivo* by silencing miR-17 and miR-19a was observed.^139^ Furthermore, overexpression of miR-17∼92 was also associated with upregulation of MYC expression.^138^ Likewise, overexpression of miR-106b in precursor cells promoted SHH pathway activation.^142^ miR-106b also positively regulated Gli2 transcription to promote granule cell expansion.^144^ Moreover, low expression of miR-466f could affect the Vegfa/Nrp2 pathway, thus sustaining the mesenchymal phenotype of SHH MB stem cells.^145^ miR-218 expression was decreased in the SHH and group 3 MBs.^125^^,^^126^ Notably, decreased expression levels of miR-182 and miR-183 in SHH MB compared to non-SHH MB were also observed.^119^ A recent study identified 19 miRNAs that exhibited MB group 4-specific expression compared to the other subgroups.^146^

### lncRNAs and circRNAs in MBs

**Figure 1:**
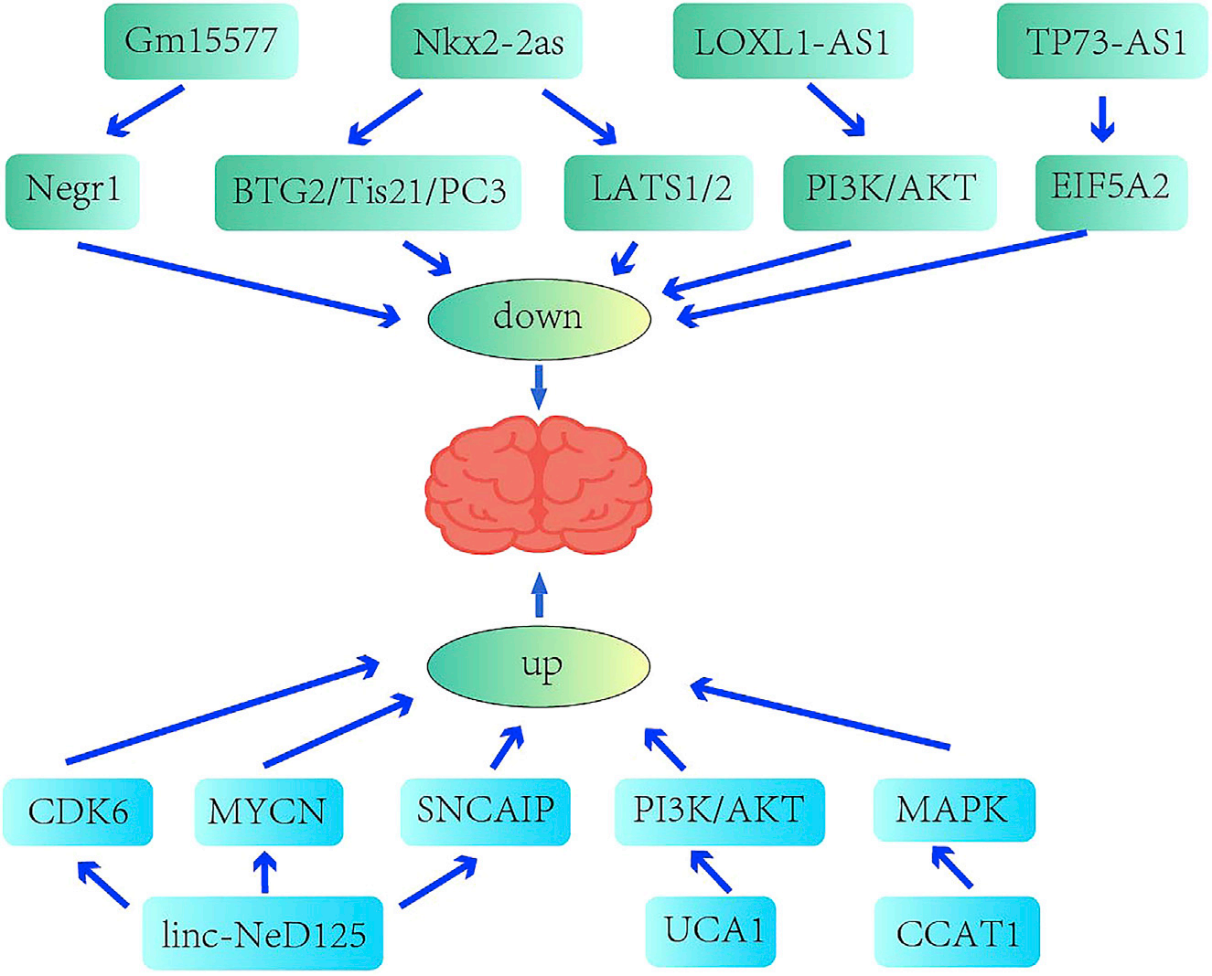
Roles of lncRNAs in medulloblastoma

Recent studies have revealed that the expression of many lncRNAs are dysregulated in MBs (Figure 1). lncRNA Gm15577 was found to be specifically expressed in the mouse cerebellum in a developmentally regulated manner by targeting Negr1, which had a distinct expression pattern in MB patients from normal patients. Gm15577 might be associated with tumorigenesis of MBs.^18^ Overexpression of lncRNA CRNDE promoted tumor growth both *in vitro* and *in vivo* by arresting cell cycle progression and inhibiting apoptosis.^19^ One study reported that interference with lncRNA SPRY4-IT1 expression inhibited cell proliferation, invasion, and metastasis of MB cells.^147^ In addition, lncRNA linc-NeD125 was observed to be significantly overexpressed in group 4 MBs. Further *in vitro* experiments proved that linc-NeD125 acted as a competing endogenous RNA (ceRNA) by sequestering three miRNAs, that is, miR-19a-3p, miR-19b-3p, and miR-106a-5p, which derepressed the major driver factors CDK6, MYCN, and SNCAIP in group 4 MBs.^21^ Thus, downregulation of linc-NeD125 expression inhibited group 4 cell proliferation. In addition, it was proven that ectopic expression of linc-NeD125 also attenuated cell proliferation, migration, and invasion in aggressive group 3 MBs.^21^

Downregulation of the lncRNA Nkx2-2as was found to contribute to tumorigenesis when SHH signaling in cerebellar granule cells was constitutively activated. Nkx2-2as functions as a ceRNA to sequester miR-103/107 and miR-548 m, and it downregulated the tumor suppressors BTG2/Tis21/PC3 and LATS1/2, promoting tumor growth both *in vitro* and *in vivo*.^22^ Elevated expression of the lncRNAs UCA1 and CCAT1 was also observed in MB specimens, and knockdown of UCA1 and CCAT1 significantly suppressed MB cell proliferation and migration.^23^^,^^24^ Gao et al.^25^ provided both *in vitro* and *in vivo* evidence that downregulation of the lncRNA LOXL1-AS1 impaired tumor cell growth and migration through the PI3K/AKT pathway, displaying a potent pro-oncogenic function in MB.

One group reported that the lncRNA TP73-AS1 was clinically relevant in MB and could promote the survival, migration, and proliferation of MB cells *in vitro* and tumorigenicity *in vivo*.^26^ Mechanistically, TP73-AS1 was further found to positively regulate EIF5A2 expression by sponging miR-494-3p.^27^ Moreover, knockdown of the lncRNA HOTAIR inhibited MB cell proliferation, tumor growth, migration, and invasion and promoted cell apoptosis via regulation of the miR-1/miR-206-YY1 axis and epithelial-to-mesenchymal transition (EMT).^29^ Likewise, depletion of CRNDE also suppressed MB cell proliferation, apoptosis, migration, invasion, and chemosensitivity to cisplatin by binding to miR-29c-3p.^20^ Based on next-generation sequencing for discovery and qRT-PCR for validation, circ-SKA3 and circ-DTL were proven to be overexpressed in MB tissues compared with normal cerebellar tissues, whereas circ-CRTAM, circ-MAP3K5, circ-RIMS1-1, and circ-FLT3-1 were significantly downregulated.^148^ Downregulation of circ-SKA3 and circ-DTL was further confirmed to suppress MB cell proliferation, migration, and invasion by regulating the expression of host genes.^148^

## EPN

As the third most common pediatric brain tumor, EPN mainly occurs in children under 5 years of age.^3^^,^^149^ This devastating disease was thought to originate from ependymal cells located in the lining of ventricular surfaces in the brain, and it occurs most commonly at the midline or lateral compartments of the posterior fossa in children.^150^ The current therapeutic strategy for pediatric EPN remains maximal safe surgical resection of the tumor combined with radiotherapy; however, this treatment seriously affects the growth and development of pediatric patients. Based on the WHO classification of CNS tumors, EPNs have been traditionally subdivided into distinct entities and histological variants.^151^ However, the utility of the WHO grade-based risk classification is controversial and inconclusive due to its limited predictive power.^149^^,^^152^^,^^153^ Recent advances in the biological characterization of ependymal tumors have distinguished nine molecular subgroups that appear to reflect more precise clinicopathological and molecular features, with three occurring in each anatomic compartment, exhibiting the potential for guiding therapeutic decisions.^154^ Therefore, the discovery of new molecular biomarkers and potential mechanisms has a great impact on the understanding of EPN.

### miRNAs in EPN

It is encouraging to note that a number of miRNAs have been identified to associate with the biology of ependymal tumors and serve as potential candidates for molecular therapeutic targets. Analysis of microarray data showed an upregulation of miR-34b, miR-34c, miR-200a, miR-200b, and miR-483 in EPN, while miR-124a, miR-137, miR-138, miR-193b, and miR-181d appeared to be downregulated in EPN samples.^71^ Costa et al.^155^ identified 28 miRNAs differentially expressed in EPNs compared to normal controls via miRNA expression profiling. miR-34a and miR-135a were further verified to be overexpressed, while miR-485-5p was downregulated. Another study also identified that the miR-135a-3p, miR-137, miR-17-5p, miR-181d, and let-7d-5p were upregulated in EPNs.^156^ Low expression of miR-10a and high expression of miR-10b and miR-29a in EPN were also validated by qRT-PCR.^157^

Specifically, miR-17-5p, miR-19a-3p, miR-106b-5p, miR-124-3p, and miR-203a were shown to be differentially expressed between grade II and III EPNs.^156^^,^^158^^,^^159^ These miRNAs were overexpressed in posterior fossa EPNs, including miR-106-b-5p and miR-19a-3p.^158^ Moreover, miR-203, miR-17-5p, miR-124-3p, miR-192-5p, miR-221-3p, miR-222-3p, miR-326, miR-371a-5p, and miR-520g-3p were significantly correlated with tumor relapse.^155^^,^^158^, ^159^, ^160^ Furthermore, let-7d, miR-596, miR-367, miR-203, miR-17-5p, miR-124-3p, miR-203, miR-15a, and miR-24-1 were found to be associated with overall survival.^73^^,^^155^^,^^158^^,^^159^^,^^161^ The relationship between miRNA expression and tumor treatment response has also been addressed. For example, miR-135a and miR-146b were found to be associated with a low-response phenotype, which could lead to recurrence of the tumor.^157^

Mechanistically, through a study on pediatric spine EPNs by Lourdusamy et al.,^162^ miR-10b and miR-10a were found to be upregulated and targeted chromatin modification genes. miR-124, a tumor suppressor, was downregulated in pediatric spine EPNs and repressed cell-cell communication and genes involved in metabolic processes. Yang et al.^163^ performed a miRNA-mRNA network analysis and identified six crucial miRNAs, including miR-34a-5p, miR-449a, miR-106a-5p, miR-124-3p, miR-128-3p, and miR-330-3p, that might be utilized as biomarkers and potential therapeutic targets for EPN. Based on miRNA-mRNA covariation and sequence-based target predictions, miR-29a/c was identified as a regulator of LAMA2, revealing a key mechanism for molecular pathogenesis.^164^ In addition, miR-495-3p and miR-299 were identified by qRT-PCR and had CD44 positively co-regulated potential targets, such as VEGFA and CSF1, which are associated with tumor progression and a worse prognosis.^165^ Two oncogenic molecules, miR-15a and miR-24-1, were also identified in coexpression networks to regulate expression of CYP11B1, KRT33B, RUNX1T1, SIK1, MAP3K4, MLANA, and SFRP5 via a weighted gene coexpression network approach.^166^

### lncRNAs in EPN

**Figure 2:**
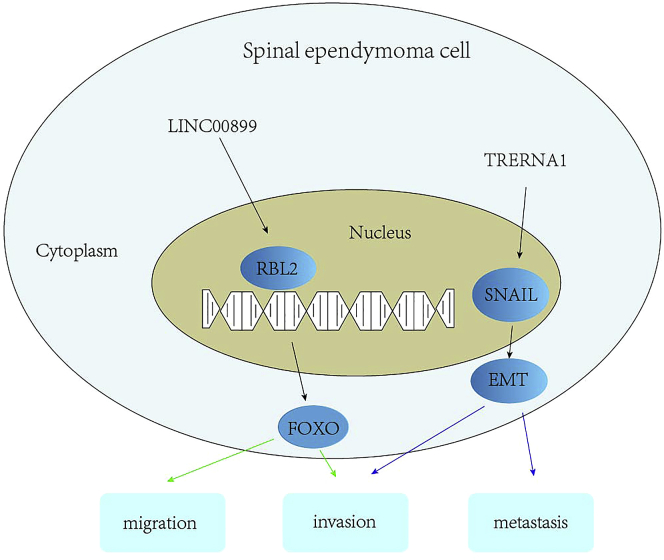
Roles of lncRNAs in ependymoma

The lncRNA LINC00899 was observed to be upregulated in spinal EPN samples. Further *in vitro* experiments verified the anti-oncogenic effects of downregulated LINC00899, which inhibited spinal EPN cell invasion and migration via the RBL2-dependent FoxO pathway.^38^ The lncRNA TRERNA1, which regulates the expression of the EMT master transcription factor Snail, was significantly overexpressed in intracranial subgroups compared to normal brain. TRERNA1 upregulation was found to be associated with higher proliferative indices and shorter progression-free survival.^39^ Using a genome-wide methylome analysis approach, Wang et al.^167^ identified lncRNA signatures associated with tumor histological characteristics based on the methylation status of lncRNA promoters. The lncRNA LINC00052 exhibited the highest importance value in the classification of spinal EPNs. Another study found low expression of the lncRNA HOTAIR in EPN, which is also known as metastasis-associated lncRNA.^28^ Taken together, lncRNAs participate in EPN progression (Figure 2).

## Diffuse intrinsic pontine glioma (DIPG)

DIPGs are a devastating spectrum of disease with no effective cures, although a myriad of treatments have been studied in hundreds of clinical trials.^168^, ^169^, ^170^ As a subtype of advanced grade gliomas that originates in the pons and spreads to other parts of the brainstem, almost all confirmed patients will die of this disease within 2 years from the time of their initial diagnosis.^169^^,^^171^ To date, no chemotherapeutic strategy has been shown to improve the prognosis of DIPGs, and the predominant method of treatment for children with newly diagnosed DIPG remains focal radiotherapy to the pons.^172^^,^^173^ Due to the tumor location, great advances in neurosurgical techniques have allowed for DIPG surgical biopsies to be conducted safely.^169^^,^^174^^,^^175^ A combination of genomic profiling and drug efficacy testing will result in a better understanding of DIPG biology and assist in drug development.

According to the current research, DIPG has specific driver mutations that could promote invasion. A specific point mutation in one of the histone 3 genes, including *H3.3* (*H3F3A*) or *H3.1* (*HIST1H3B*), appears in most DIPG cases (80%) and causes lysine 27 on the amino-terminal tail to be replaced with a methionine (H3K27M).^176^, ^177^, ^178^ In fact, mutations in *HIST1H3B* resulted in a better prognosis than mutations in *H3F3A*.^179^ In addition, several genomic alterations or amplifications are observed in *Tp53*, activin A receptor type I (*ACVR1*), and platelet-derived growth factor receptor alpha (*PDGFRA*) in DIPGs.^179^

### miRNAs in DIPG

Related studies have proven that ncRNAs have an important impact on the pathogenesis of DIPGs. miR-129-2 was found to be downregulated by hypermethylation of miR-129-2 promoter in DIPGs, leading to overexpression of NG2, which was demonstrated to contribute to the neoplastic transformation of glioma cells.^180^ A bioinformatics analysis of microarray data demonstrated 27 altered miRNAs associated with DIPG and built a miRNA-target regulatory network consisting of 141 miRNA-target gene pairs. Moreover, miR-26b, which interacts with TFAP2 to a higher degree in the transcription factor (TF)-miRNA-target gene regulatory network, might have a critical role in the tumorigenesis of DIPG.^181^

### lncRNAs in DIPG

Using expression profile analysis, Liu et al.^182^ also identified some novel differentially expressed lncRNAs in DIPG, including AF086127, AF086217, AF086391, AF119852, AK021535, AK022370, AL050068, BC012548, and BC041658. These lncRNAs are significantly correlated with DIPG survival and have great potential as diagnostic or prognostic biomarkers. Continuous exploration of specific biomarkers will provide new possibilities for humans to understand the underlying mechanisms of DIPG.

## Craniopharyngioma (CP)

Childhood-onset CPs are rare nonglial tumors of the sellar region originating from remnants of the craniopharyngeal duct epithelium with low-grade histological malignancy.^183^^,^^184^ CPs are traditionally classified into two histological subtypes, adamantinomatous CP (ACP) and papillary CP (PCP), which exhibit distinct genetic features and age distributions.^185^, ^186^, ^187^ Despite favorable survival outcomes, the quality of life of these pediatric patients is frequently impaired due to the severe sequelae of this disease.^188^, ^189^, ^190^ Thus, novel insights into the molecular pathogenesis of CPs will help develop new treatments targeting pathogenic pathways, thereby decreasing or eliminating adverse effects.

### miRNAs in CP

Based on miRNA expression analysis, overexpression of miR-150 and decreased expression of let-7a, miR-16, miR-15a, miR-23b, miR-24-2, miR-141, miR-143, miR-145, and miR-449 were observed in ACPs.^191^ Further *in silico* analysis indicated that miR-150, miR-23b, miR-24-2, miR-141, and miR-449 could regulate CTNNB1 expression through the Wnt signaling pathway, suggesting important roles in ACP tumorigenesis.^191^ Another extensive miRNA expression analysis demonstrated that downregulation of miR-132 appeared to be an indicator of aggressiveness and might contribute to EMT.^192^ The role of ncRNAs in CPs is understudied and research is still needed to explore their underlying biological and pathogenic mechanisms.

## miRNAs and lncRNA in ATRTs

Most ATRTs are characterized by genomic alterations in SMARCB1 or, to a less extent, SMARCA4 of the SWItch/sucrose nonfermentable chromatin remodeling complex.^193^^,^^194^ To identify new possible therapeutic targets of ATRT, Sredni et al.^195^ demonstrated dysregulated expression of miR-221/222 in ATRT, and their upregulation contributed to oncogenesis and development of ATRT by p27Kip1 downregulation. Both miRNAs regulate the expression level of the target gene SUN2, which is a tumor suppressor and accelerates cell proliferation and tumor malignancy both *in vitro* and *in vivo*.^196^ Birks et al.^71^ also found that miR-129, miR-142-5p, and miR-25 were differentially expressed in five pediatric brain tumor types, including ATRT, compared to normal tissue controls. In the same study, the upregulated miRNAs in ATRTs, including miR-520b, miR-629, miR-221, miR-448, and miR-373, and the downregulated miRNAs, including miR-140, miR-let-7b, miR-139, miR-153, and miR-376, were also revealed. Deletion of miRNAs let-7a3 and let-7b was found to partially contribute to the overexpression of the oncoprotein HMGA2 in ATRT tissues. Upregulation of let-7 miRNA or knockdown of HMGA2 could also inhibit rhabdoid tumor cell proliferation, colony formation, and invasion.^197^ In addition, it was demonstrated that miR-142-3p was downregulated in stem-like ATRT cells (ATRT-CD133^+^), and that its lower expression promoted tumor growth and invasive, radioresistant, and stem-like capacities. Notably, therapeutic delivery of miR-142-3p in ATRT cells effectively reduced its lethality and prolonged survival time in orthotropic-transplanted immunocompromised mice.^198^ More recently, tumor-associated mesenchymal stem cells were observed to secrete miR-155-enriched exosomes, and the abundant expression of exosomal miR-155 could mediate ATRT tumor migration through downregulation of the tumor suppressor SMARCA4.^199^ Moreover, transcriptome analysis also indicated significantly higher expression of the lncRNA HOTAIR and its associated protein-coding gene *HOXC* in ATRT tissues, although the underlying mechanism needs further investigation.^28^

## Glioblastoma (GBM)

GBM (WHO grade IV) is the most common and malignant primary brain tumor. It occurs more frequently in adults and only accounts for approximately 8%–12% of all CNS tumors in children.^200^^,^^201^ This neoplasm is characterized by rapid diffuse and infiltrative growth and a high level of cellular heterogeneity leading to therapeutic resistance.^202^ Thus, despite the multimodal treatment procedure composed of surgical intervention, radiotherapy, and temozolomide-based chemotherapy, the overall survival of these patients is still unsatisfactory with a median survival of 15 months,^203^^,^^204^ Thus, it is urgent to discover novel therapeutic strategies.^205^

### miRNAs in GBM

During recent years, large-scale research efforts have been made to unveil the roles of many ncRNAs in adult GBM onset, progression, invasiveness, and recurrence. However, because of their rarity, research on ncRNAs in pediatric GBM is still scant.^67^^,^^206^ As one of the most extensively investigated miRNAs, oncogenic miR-21 is significantly overexpressed in GBM and inversely correlated with GBM survival. Many specific molecules, including HNPRK, TAP63, PDCD4, p53, and transforming growth factor (TGF)-β and the mitochondrial apoptotic pathway regulated by miR-21, have also been validated to play a critically important role in different aspects of tumor pathogenesis.^205^^,^^207^^,^^208^ Another extensive investigation showed that miR-221 and miR-222 are among the most frequent and significantly overexpressed miRNAs in GBMs. High levels of miR-221/222 promote cell proliferation, cell cycle progression, migration, and invasion and inhibit cell apoptosis by directly targeting p27, p57, astrocytic connexin Cx43, PTPμ, TIMP3, and p53 upregulated modulator of apoptosis (PUMA).^209^, ^210^, ^211^, ^212^, ^213^, ^214^

It is known that p27, p57, Cx43, and PUMA proteins are encoded by *PSMD9*, *CDKN1C*, *GJA1*, and Bcl-2-binding component 3 (*BBC3*) genes, respectively. With respect to specific pediatric GBM patients, miR-129, miR-142-5p, and miR-25 were found to display differential expression compared to normal tissue controls.^71^ Another genome-wide microarray analysis comparing pediatric GBM patients with controls demonstrated differential expression of 266 miRNAs, of which 55 were upregulated and 71 were downregulated. Upregulated miRNAs, including miR-10b, miR-891a, miR-182, miR-155, miR-424, and miR-130b and downregulated miRNAs, including miR-138, miR-7, and miR-129, were further validated by qRT-PCR. In regard to the expression patterns of clustered miRNAs, all miR-17/92 and miR-106b/25 cluster miRNAs were upregulated, while most 14q32 cluster miRNAs were downregulated and associated with patient survival. H3F3A mutation-associated miRNA expression profiles showed that miR-15a, miR-424, miR-30e, and miR-378c were more highly expressed in H3F3A mutants than in the wild-type. A list of TP53 mutation-specific miRNAs in pediatric GBM patients was also identified. Further comparisons of miRNA expression profiles of pediatric and adult GBMs were conducted, and specially expressed miRNAs related to pediatric GBM might be associated with PDGFR-β, regulation of nuclear SMAD2/3 signaling, calcineurin, ErBB1 signaling, and cdc42 signaling pathways.^215^

In addition, new molecular characteristics of pediatric and adult high-grade gliomas were revealed to support their biological differences. For example, the miR-17-92 cluster was found to be upregulated in pediatric high-grade gliomas, where it controlled cell proliferation and targeted tumor suppressors such as PTEN.^216^ Giunti et al.^217^ also examined the miRNA expression profile of pediatric GBM and demonstrated that miR-137, miR-490, miR-876-3p, miR-876-5p, and miR-448 were downregulated and miR-501-3p was upregulated. The association of each of the identified differentially expressed miRNAs with NUCKS1 deserves further investigation. Moreover, overexpression of miR-487b in a pediatric glioma cell line (KNS42), which was established from a 16-year-old child with GBM, downregulated PROM1 and Nestin. This resulted in the inhibition of colony formation, whereas cell growth, proliferation, sensitivity to temozolomide, migration, and invasion were not affected.^218^ Using a predictive analysis approach for pediatric and adult high-grade glioma, Liu et al.^219^ also screened 12 microarrays and identified miR-10a, miR-10b, and miR-139 as having common differences in glioma.

### lncRNAs in GBM

The lncRNA MALAT1 has been shown to be overexpressed in GBM and is associated with worse outcomes for GBM patients. Mechanistically, MALAT1 serves as a “molecular sponge” and can modulate the activity of multiple miRNAs, including miR-106-5p, miR-144-3p, miR-211, miR-203, miR-155, and miR-199a. The modulation of these miRNAs has an important impact on the pathogenesis and development of tumors, including GBM.^40^, ^41^, ^42^, ^43^, ^44^ The lncRNA HOTAIR has been found to be highly expressed in several types of pediatric brain tumors.^28^ HOTAIR also functions as a sponge miRNA, and its depletion inhibits the malignant biological behaviors of GBM.^30^, ^31^, ^32^, ^33^, ^34^, ^35^, ^36^, ^37^ One study indicated dysregulation of HOX genes and HOTAIR in pediatric GBMs.^28^ Another study confirmed that HOXA9 directly binds with the HOTAIR promoter in adult and pediatric glioma-derived cell lines.^33^ The lncRNA DGCR5 was also found to suppress EMT in pediatric primary GBM cells and might serve as a prognostic biomarker.^45^

### circRNAs in GBM

As a new research hotspot, circRNAs have been increasingly valued by researchers in GBM (Table 2). circ-FBXW7 encodes a novel 21-kDa protein, FBXW7-185aa, which regulates cell proliferation and the cell cycle. Knockdown of FBXW7-185aa promoted malignant phenotypes *in vitro* and *in vivo*.^46^ Both circ-SHPRH and its encoded protein SHPRH-146aa were found to be downregulated in GBM. The overexpression of SHPRH-146aa reduced malignant behavior and tumorigenicity *in vitro* and *in vivo*.^47^ circSMARCA5 was significantly downregulated in GBM and associated with tumor progression. Overexpression of circSMARCA5 inhibited the migration of GBM cells by regulating a molecular axis that involves the splicing factors SRSF1/SRSF3/PTB.^48^ Moreover, circSMARCA5 was found to be an upstream regulator of the proangiogenic-to-antiangiogenic VEGFA isoform ratio.^49^ The circRNA hsa_circ_0008344 has been studied *in vitro*, and it showed the ability to regulate GBM cell proliferation, colony formation, migration, invasion, and the cell apoptotic rate.^50^ Another oncogenic circRNA, circNT5E, has been found to act as a sponge against miR-422a in GBM tumorigenesis, controlling many pathologic processes, such as cell proliferation, migration, and invasion.^51^ circPINTexon2 has been observed to produce a peptide named PINT87aa, which suppresses GBM cell proliferation *in vitro* and *in vivo*.^52^ Wang et al.^53^ also indicated that eIF4A3-induced circMMP9 could promote GBM cell proliferation, invasion, and metastasis via the miR-124 signaling pathway. Elevated circ_0029426 was observed in GBM tissues and could strongly promote cell proliferation, migration, and invasion and inhibit cell apoptosis by sponging miR-197.^54^ Moreover, the circ_0001946/miR-671-5p/CDR1 pathway modulates the development of GBM.^220^ Upregulation of circ_0074027 also promoted GBM cell growth and invasion by regulating the miR-518a-5p/IL17RD signaling pathway.^55^ hsa_circ_0067934 was found to be upregulated in GBM and promoted cancer cell proliferation and metastasis via upregulation of the PI3K-AKT pathway.^56^

circ_0001730 has been found to promote GBM cell proliferation and invasion via the miR-326/Wnt7B axis.^57^ circMTO1 has been reported to inhibit GBM cell proliferation via the miR-92/WWOX signaling pathway.^58^ Impaired expression of circ-AKT3 contributed to GBM tumorigenesis.^59^ Moreover, circ-PITX1 could act as a ceRNA to promote the progression of GBM by regulating the miR-379-5p/MAP3K2 axis.^60^ Similarly, circFOXO3 was discovered to promote GBM progression by sponging both miR-138-5p and miR-432-5p to regulate the expression of NFAT5.^61^ Furthermore, overexpression of circ-0001801 contributed to GBM cell proliferation, migration, invasion, and EMT by modulating the miR-628-5p and HMGB3 axes.^62^ circ-EPB41L5 was also shown to play a striking role in the progression of GBM via regulation of the miR-19a/EPB41L5/p-AKT regulatory axis.^63^ Most recently, circENTPD7 was found to promote GBM cell proliferation and motility by regulating miR-101-3p/ROS1.^64^ However, the expression of these circRNAs needs further confirmation in GBM in the pediatric setting.

## Conclusions and future perspectives

The advancement of bioinformatics technology has greatly facilitated the identification of a great number of abnormally expressed ncRNAs in different types of tumors. Currently, there is consensus that ncRNAs play important roles in gene regulatory networks and hold great potential as therapeutic targets. As discussed above, accumulating evidence has clearly supported the involvement of ncRNAs in pathogenesis as oncogenes or tumor suppressors in pediatric neuro-oncology. First, ncRNAs can better enable researchers to discover molecular markers that help with tumor classification and patient risk stratification combined with other biological characteristics, thereby assisting in standardizing the selection and treatment plans.^221^ The molecules that are involved in tumor occurrence and development, as well as the regulation of response to therapy, can be further employed to assess therapeutic effects and screen potential patients who can significantly benefit from other therapeutic opportunities, such as immunotherapy and gene therapy.

In addition, treatments targeting these aberrantly expressed ncRNAs are a promising approach to improve the outcomes of pediatric patients with CNS tumors. Despite the great potential, the nonspecificity of ncRNA targets has to be taken into account, and the delivery method for ncRNAs should be optimized to be effective and nontoxic.^221^^,^^222^ Future studies must address these issues to drive ncRNA-based therapeutic development. Without a doubt, further intensified exploration is needed to discover additional ncRNAs with crucial biological functions and deepen the understanding of these molecules as therapeutic targets in the management of pediatric CNS tumors. In summary, ncRNA studies continue to provide new insights into pediatric neuro-oncology biology. Although this field faces many challenges and significant efforts are still required, clinical applications of ncRNAs for pediatric CNS tumors will drastically change the medical practice in the foreseeable future.
